# The complete chloroplast genome sequence of *Paphiopedilum purpuratum* (Orchidaceae)

**DOI:** 10.1080/23802359.2019.1688106

**Published:** 2019-11-08

**Authors:** Xian-De Chen, Dong-Hui Peng, Si-Ren Lan, Jun Chen, Wen-Qun Fu

**Affiliations:** a Fujian Ornamental Plant Germplasm Resources Innovation and Engineering Application Research Center, Fuzhou, China;; b Key Laboratory of National Forestry and Grassland Administration for Orchid Conservation and Utilization at College of Landscape Architecture, Fujian Agriculture and Forestry University, Fuzhou, China;; c College of Biological Science and Biotechnology, Minnan Normal University, Zhangzhou, China

**Keywords:** Chloroplast genome, phylogenetic, Illumina sequencing, *Paphiopedilum purpuratum*

## Abstract

*Paphiopedilum purpuratum*, an endangered terrestrial orchid distributed in southwestern and south of China. In this study, the complete chloroplast genome (cpDNA) sequence of *P. purpuratum* was determined from Illumina pair-end sequencing data. With a total length of 158,459 bp in length and includes two inverted repeat regions (IRs) of 34,484 bp each, which were separated by a large single-copy region (LSC) 88,022 bp and a small single-copy region (SSC) 1,469 bp. The chloroplast genome contained 126 genes, including 74 protein conding genes,38 tRNA genes, and 8 rRNA genes. Phylogenetic analysis indicated that *P. purpuratum, P. dianthum, P. niveum, P. delenatii,* and *P. armeniacum* cluster together, placed them within genus *Paphiopedilum*. The complete chloroplast genome sequence of *P. purpuratum* will provide a useful resource for the evolutionary biology study of phylogenetic studies in Orchidaceae.

*Paphiopedilum purpuratum* is an Endangered plant that belongs to *Paphiopedilum* genus which contains 88 species (Luo et al. 2003), mainly distributed in southwestern and south China, including Fujian, Guangdong, Guangxi, Hainan, Hongkong and Yunnan (Liu et al. 2009; You et al. 2009). The destruction of resources is extremely serious and on the verge of extinction. The present study assembled and characterized the chloroplast genome of *P. purpuratum* as a resource for evolution and breeding research.

Fresh Leaf sample of *P. purpuratum* was acquired from Mount Wushan (N24°05′, E116°59′), Zhangzhou City, Fujian Province of China, and voucher specimen deposited at Herbarium of College of Forestry, Fujian Agriculture and Forestry University (specimen code 2006001). The total genomic DNA was extracted from fresh leaves using a modified CTAB method (Doyle and Doyle 1987) and sequenced based on the Illumina pair-end technology. The clean reads were firstly aligned to *P. dianthum* (GenBank accession No. NC036958) and then assembled into contigs in the software CLC Genomics Workbench v8.0 (CLC Bio, Aarhus, Denmark). The assembled chloroplast genome was annotated using OGDRAW, and the annotation was corrected using Geneious (Kearse et al. 2012). The physical map of the new chloroplast genome was generated using OGDRAW (Lohse et al. 2013). The accurate new annotated complete chloroplast genome was submitted to GenBank with accession number MN535015.

The complete chloroplast genome of *P. purpuratum is* 158,459 base pairs (bp) in length, containing a large single-copy (LSC) region of 88,022 bp, a small single-copy (SSC) region of 1469 bp, and two inverted repeat (IR) regions of 34,484 bp. Complete chloroplast genome contains 126 genes, there were 74 protein-coding genes, 38 tRNA genes, and 8 rRNA genes. The overall GC-content of the whole chloroplast is 35.4%, while the corresponding values of the LSC, SSC, and IR regions are 34.5, 26.6 and 39.2%, respectively. Besides, six unique pseudogenes were annotated. The phylogenetic analysis was carried out with *P. purpuratum* and 14 other complete cp genome of species from Orchidaceae (*Neuwiedia zollingeri* var. *singapureana*, *Apostasia odorata*, *Apostasia wallichii*, *Cypripedium macranthos*, *Vanilla aphylla*, *Vanilla planifolia*, *Vanilla pompona*, *Cypripedium formosanum*, *Phragmipedium longifolium*, *Paphiopedilum armeniacum*, *Paphiopedilum* micranthum, *Paphiopedilum delenatii, Paphiopedilum niveum, Paphiopedilum dianthum*). The sequences were aligned using HomBlocks pipeline (Bi et al. 2018). RAxML-HPC2 on XSEDE version 8.2.10 (Stamatakis 2014) was used to construct a maximum likelihood tree, the branch support was computed with 1000 bootstrap replicates that *P. purpuratum, P. dianthum, P. niveum, P. delenatii, P. micranthum* and *P. armeniacum* cluster together, placed them within genus *Paphiopedilum* (Figure 1).

**Figure 1.**
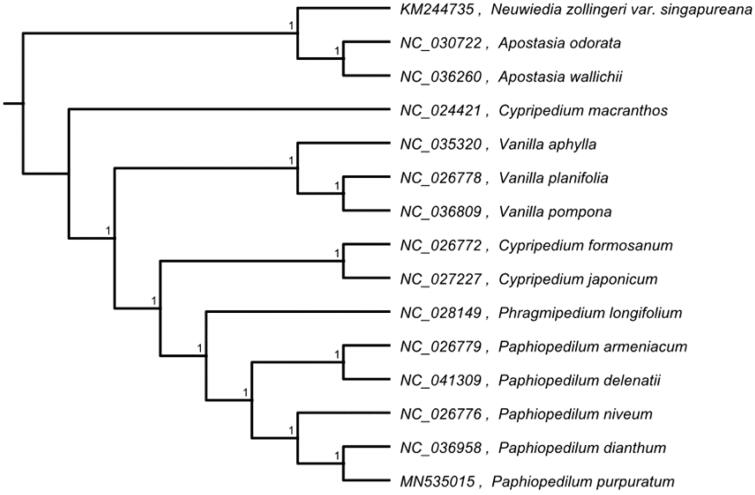
Maximum-likelihood tree based on the complete cp genome sequences of 15 species from the Orchidaceae. Shown next to the nodes are bootstrap support values based on 1000 replicates.
